# The resistance-compliance product of the pulmonary circulation varies in health and pulmonary vascular disease

**DOI:** 10.14814/phy2.12363

**Published:** 2015-04-22

**Authors:** Charaka Hadinnapola, Qiuju Li, Li Su, Joanna Pepke-Zaba, Mark Toshner

**Affiliations:** 1 Pulmonary Vascular Diseases Unit, Papworth Hospital NHS Foundation TrustPapworth Everard, Cambridge, UK; 2 MRC Biostatistics Unit, Cambridge Institute of Public Health, Cambridge Biomedical CampusRobinson Way, Cambridge, UK

**Keywords:** Pulmonary arterial hypertension, pulmonary artery compliance, RC time

## Abstract

Pulmonary vascular resistance (PVR) is traditionally used to describe pulmonary hemodynamic characteristics. However, it does not take into account pulmonary artery compliance (Ca) or pulsatile flow. The product of PVR and Ca is known as RC time. Previous studies assert that the PVR-Ca relationship is fixed and RC time is constant between health and disease states. We hypothesized that RC time was not constant in health and pulmonary vascular disease. Right heart catheterizations performed in Papworth Hospital over a 6 year period were analyzed. Subjects were divided into those with normal pulmonary hemodynamics (NPH group; *n* = 156) and pulmonary arterial hypertension (PAH group; *n* = 717). RC time and the right ventricle (RV) oscillatory power fraction were calculated. RC time for the NPH group (0.47 ± 0.13 sec) is significantly lower than the PAH group (0.56 ± 0.16 sec; *P* < 0.0001). The RV oscillatory power fraction is lower in the NPH group (*P* < 0.0001). RC time correlates inversely with the RV oscillatory power fraction in each group. We conclude, there is an inverse relationship between PVR and Ca, however, this relationship is not always fixed. Consequently, RC time is significantly lower in health compared to disease with elevated pulmonary artery pressures. PAH leads to a decrease in cardiac efficiency.

## Introduction

Classically, pulmonary hypertension has been defined by mean pulmonary artery pressure (mPAP) and hemodynamic assessment of patients has focused on pulmonary vascular resistance (PVR), particularly in therapeutic trials. Despite this mPAP and PVR do not correlate well with clinical outcomes, whereas measures of right ventricular (RV) function, such as the cardiac index (CI) and right atrial pressure, generally outperform pulmonary vascular parameters (Swiston et al. 2010). This is postulated to be due to the failure of PVR to accurately describe afterload and its impact on the right ventricle. Compliance (Ca) is a component of afterload that can potentially be derived from commonly used fluid-filled right heart catheter-based studies. Mahapatra et al. have demonstrated the importance of Ca by showing a negative correlation between Ca and mortality in idiopathic pulmonary arterial hypertension (IPAH) (Mahapatra et al. 2006).

Many studies have reported an inverse relationship between PVR and Ca (Reuben 1971; Lankhaar et al. 2006, 2008; Saouti et al. 2009; Bonderman et al. 2011; de Perrot et al. 2011; Tedford et al. 2012; Hilde et al. 2013; Pagnamenta et al. 2013). This relationship has additionally been reported to be fixed. As a consequence, the product of PVR and Ca (known as RC time) is thought to be constant. RC time reflects the time constant of the pulmonary circulation (Reuben 1971). Previous studies have asserted that RC time is fixed in health, disease and following therapeutic interventions for pulmonary arterial hypertension (Lankhaar et al. 2006, 2008; Bonderman et al. 2011; de Perrot et al. 2011; Skoro-Sajer et al. 2014).

A constant pulmonary RC time implies that compliance cannot change independently of resistance and therefore it is not an important independent determinant of outcome. These conclusions are derived from studies that have all been in small cohorts and not powered to investigate differences in RC time. Furthermore, the only study to implicitly compare the RC time of patients with IPAH and chronic thromboembolic pulmonary hypertension (CTEPH) with a comparator group with no pulmonary hypertension assumed that pulmonary capillary wedge pressure (PCWP) was negligible (Lankhaar et al. 2006). As a consequence RC time was calculated using total pulmonary resistance (TPR; the ratio of mean pulmonary artery pressure (mPAP) to cardiac output). Although in patients with pulmonary arterial hypertension (PAH) the PCWP is a small fraction of mPAP, this is not the case in healthy subjects.

We aimed to establish what the RC time is in individuals with no PAH and to investigate the physiological relevance of RC time. We hypothesized that RC time is not the same in health and pulmonary vascular disease. We report here that RC time is not constant between health and pulmonary vascular disease and this may have implications for RV efficiency.

## Methods

### Subjects

The Papworth Hospital database of right heart catheterizations (RHCs) was interrogated to acquire all cases performed between 1/12/2007 and 31/12/2013. This included patients having RHCs for assessment of pulmonary hemodynamics in the context of pulmonary arterial hypertension, congenital heart disease, cardiac failure and for cardiac transplant assessment and follow up.

### Right heart catheterization

Right heart catheterizations were performed by expert cardiologists and pulmonary hypertension physicians according to international guidelines (Galie et al. 2009). Quad lumen fluid-filled Swan-Ganz catheters (Edwards Lifesciences, Irvine) connected to a Philips Haemosphere (Philips Medical Systems, Surrey, UK) were used for the procedures. All measurements were taken in the supine position and at end-expiration during one procedure. Measurements of systolic pulmonary artery pressure (sPAP), diastolic pulmonary artery pressure (dPAP), mPAP, PCWP, heart rate (HR), and cardiac output (CO) by thermodilution were recorded. Pulmonary artery pressure measurements were taken in the main pulmonary artery and mPAP was calculated as the average pressure over a given period of time.

### Analysis

**Figure 1:**
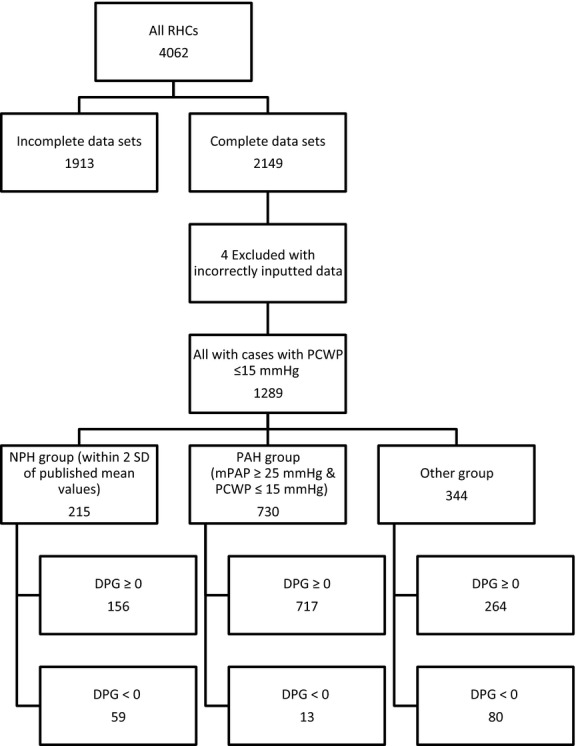
Selection criteria for NPH and PAH groups.

Right heart catheterizations with incomplete data that did not allow calculation of RC time were excluded. RHCs with negative diastolic pressure gradients (DPG) were also excluded. The dataset was divided into three groups (Fig.1): those with normal pulmonary hemodynamics (NPH group), those whose hemodynamics met the 5th World Symposium on Pulmonary Hypertension definition of pulmonary arterial hypertension (PAH group) (Hoeper et al. 2013) and those whose hemodynamics fell outside these two groups (“Other” group).

By definition all patients had a PCWP ≤15 mmHg, this minimizes the impact of left heart disease and elevated PCWPs on the RC time analysis (Tedford et al. 2012). The NPH group was selected by excluding RHCs with any hemodynamic parameter more than two standard deviations outside published mean values for healthy individuals (sPAP 20.81 ± 4.4 mmHg (mean ± SD), dPAP 8.75 ± 3.01, mPAP 13.95 ± 3.27, PCWP 7.97 ± 2.85, HR 76 ± 14 bpm and CI 4.1 ± 1.26L.min^−1^.m^−2^) (Kovacs et al. 2009). The “other” group consists of subjects whose hemodynamics did not meet the strict selection criteria for inclusion in the NPH group or the PAH group but still have a PCWP ≤15 mmHg.

We have confirmed the fidelity of the database recorded measurements by a bootstrapping validation of the pressure waveforms of a subset of 63 randomly selected subjects with PAH. Database recording was representative of pressure waveforms.

Ca was defined as the ratio of stroke volume to pulse pressure (SV/PP method) (Segers et al. 1999a; Lankhaar et al. 2006; Mahapatra et al. 2006). RC time was defined as PVR x Ca (Reuben 1971). The RV oscillatory power fraction was defined as (Saouti et al. 2010):

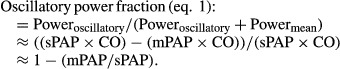
1

For this CO was defined in mL sec^−1^ and pressure in mmHg. The result is given in watts using the conversion 1W = 7500 mmHg mL sec^−1^.

### Statistical analysis

Statistical analyses were carried out using R (http://cran.r-project.org/) and Graphpad Prism (version 6.04, Graphpad Software, CA). Summary statistics (mean ± SD) were calculated and Wilcoxon rank-sum tests were performed to test the differences in patient characteristics and hemodynamics between NPH and PAH groups.

The relationship between PVR and Ca was examined using several inverse and exponential models; model fits were obtained by nonlinear least squares estimation. Data from both the NPH and PAH groups were included in the same model, but an indicator function for the PAH group was introduced to allow separate sets of parameters for the two groups. The statistical significance of the estimated parameters was suggested by the *P*-values of *t*-tests. Model selection was based on the value of “residual standard error” (smaller is better) and the number of parameters, that is, when the residual standard errors were the same for two fitted models, the one with the smaller number of parameters was preferred. Simple linear models with least squares estimation were used for examining the relationships between (1) RC time and mPAP; (2) RC time and the RV oscillatory power fraction; and (3) mPAP and sPAP. *F*-tests were used to test differences between the NPH and PAH groups.

Power and sample size calculations were carried out on Stata version 12 (StataCorp, TX), using the two-sample sampsi function. The power of referenced studies to find differences in RC time between two groups were calculated using published sample sizes, means and standard deviations as input variables and with *α* (significance level) = 0.05. While sample sizes were estimated using published means and standard deviations as input variables and with *α *= 0.05 and power = 0.9.

### Ethics

Approval for the study was given by Papworth Hospital Research and Development and a waiver of written informed subject consent for the use of anonymized retrospective data was given.

## Results

The Papworth Hospital database contained 156 RHCs which met the hemodynamic definition for inclusion in the NPH group and 717 met the 5th World Symposium on Pulmonary Hypertension definition of PAH.

### Hemodynamics and RC time

The characteristics of the NPH and PAH groups are summarized in Table1. Both groups are comprised of patients undergoing right heart catheterization for assessment and follow up of pulmonary hypertension, heart failure, congenital heart disease, and heart transplantation. The mean age of the two groups was statistically different (NPH group 53.3 ± 13.1 years, PAH group 60.4 ± 14.6 years; Wilcoxon rank-sum test *P* < 0.0001). All pulmonary hemodynamics were significantly different between the two groups.

**Table 1:** Summary of patient characteristics and hemodynamics

	NPH group	PAH group	*P*
*n*	156	717	
Age (years)	53.3 ± 13.1	60.4 ± 14.6	<0.0001
sPAP (mmHg)	24.4 ± 3.5	68.3 ± 21.4	<0.0001
dPAP (mmHg)	9.8 ± 2.4	25.4 ± 8.7	<0.0001
mPAP (mmHg)	16.0 ± 2.3	41.1 ± 12.0	<0.0001
mPAP/sPAP	0.66 ± 0.08	0.61 ± 0.06	<0.0001
PCWP (mmHg)	7.8 ± 2.3	10.5 ± 2.9	<0.0001
CO (L min^−1^)	4.6 ± 1.0	4.3 ± 1.3	0.0049
HR (bpm)	75.2 ± 13.2	78.5 ± 13.3	0.0126
PVR (Wood units)	1.9 ± 0.54	8.0 ± 4.8	<0.0001
PVR (mmHg sec mL^−1^)	0.11 ± 0.03	0.48 ± 0.29	
Ca (mL mmHg^−1^)	4.6 ± 1.7	1.6 ± 0.9	<0.0001
RC time (PVR) (sec)	0.47 ± 0.13	0.56 ± 0.16	<0.0001

Data presented as mean ± SD. *P*, compared with Wilcoxon rank-sum test. NPH group, normal pulmonary hemodynamics group; PAH group, pulmonary arterial hypertension group; sPAP, systolic pulmonary artery pressure; dPAP, diastolic pulmonary artery pressure; mPAP, mean pulmonary artery pressure; PCWP, pulmonary capillary wedge pressure; CO, cardiac output; HR, heart rate; PVR, pulmonary vascular resistance; Ca, pulmonary artery compliance; RC time (PVR), PVR-derived RC time.

**Figure 2:**
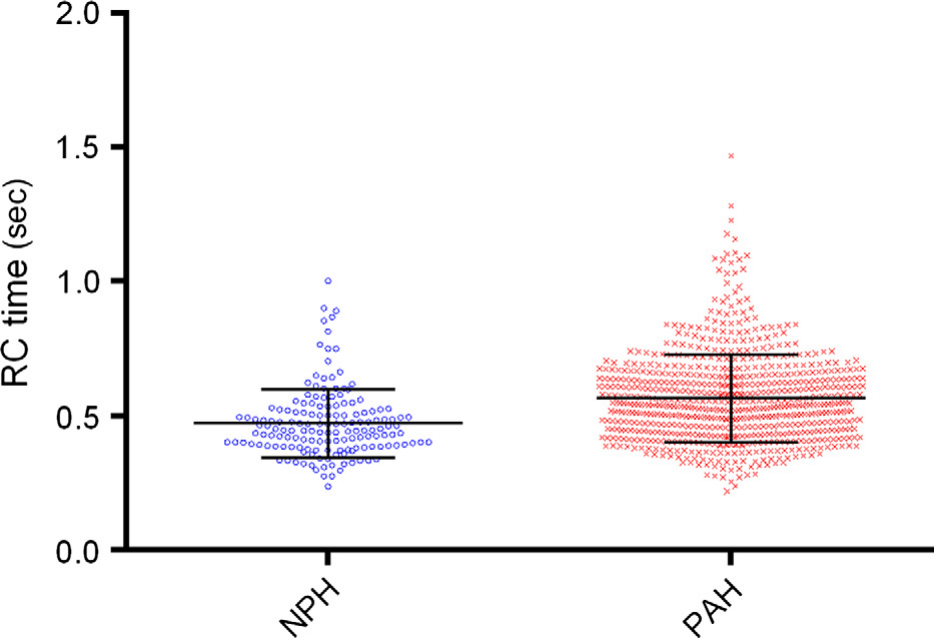
Distribution of RC time (mean ± SD).

**Figure 3:**
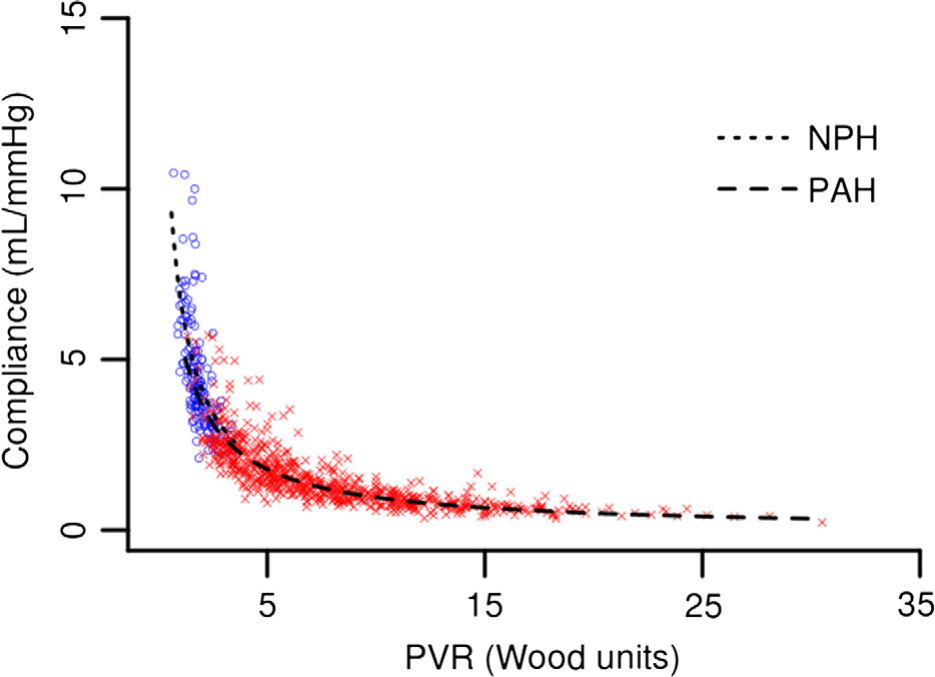
Inverse relationships between PVR and Ca. NPH: *y* = 10.4/(0.5 + PVR), PAH: *y* = 10.4/(0.8 + PVR) (*F*-test *P* = 0.0003).

RC time is significantly different between the NPH and PAH groups (0.47 ± 0.13 sec and 0.56 ± 0.16 sec, respectively; *P* < 0.0001) (Fig.2). This difference remains true if RHCs with negative DPGs are included as well. The inverse relationship between PVR and Ca proposed by Tedford et al. (with an indicator function added for the PAH group) when applied to our data was similar to our own best fitted (inverse) model (residual errors 0.697 and 0.696, respectively) (Tedford et al. 2012). Therefore, we validate and use the model proposed by Tedford et al. This relationship is significantly different between the NPH group and PAH group (*F*-test: *P* = 0.0003) (Fig.3).

**Figure 4:**
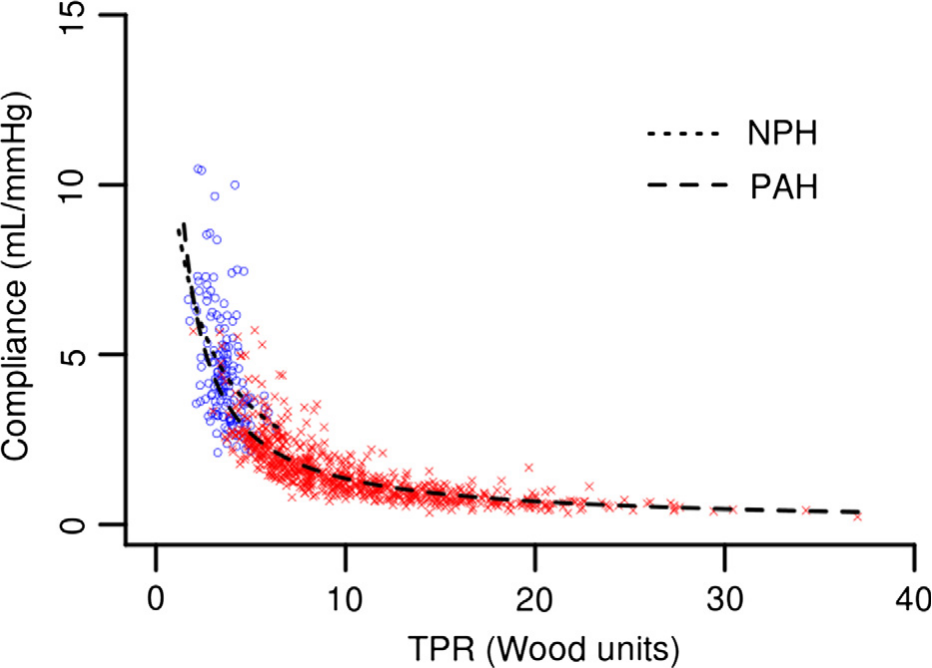
Inverse relationships between TPR and Ca. NPH: *y* = 22.1/(1.4 + TPR), PAH: *y* = 13.5/(0.1 + TPR) (*F*-test *P* < 0.0001).

When using TPR to calculate RC time; RC time for both the NPH and PAH groups are increased but remain significantly different (0.95 ± 0.32 sec and 0.79 ± 0.23 sec, respectively; *P* < 0.0001). This increase is greatest in the NPH group. The RC times calculated using TPR and PVR are significantly different for both groups (*P* < 0.0001). There remains an inverse relationship between TPR and Ca (Fig.4).

### Change in RC time with increasing mPAP

**Figure 5:**
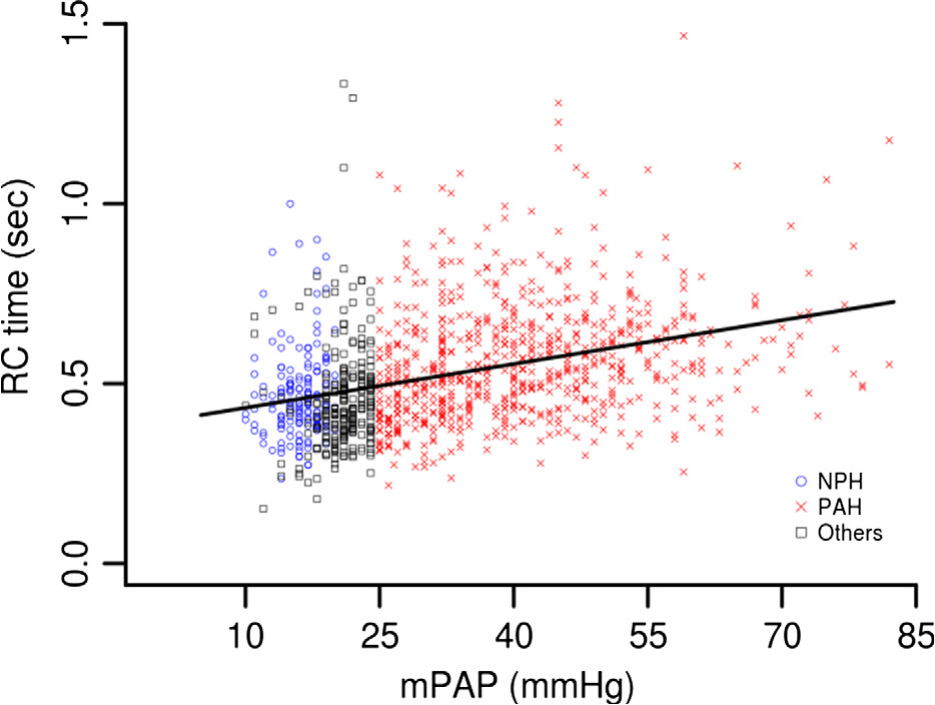
Linear fit of mPAP versus RC time. *y* = 0.39 + 0.0041 × mPAP (*P* < 0.0001, *r*^2^ = 0.128).

There is a gradual increase in RC time with increasing mPAP rather than a step increase from “normal” pressure ranges to disease states (Fig.5). The estimated slope (0.0041) and the correlation value (0.36) are significantly greater than 0 (*P* < 0.0001).

### Implications for prediction of mPAP from sPAP

**Figure 6:**
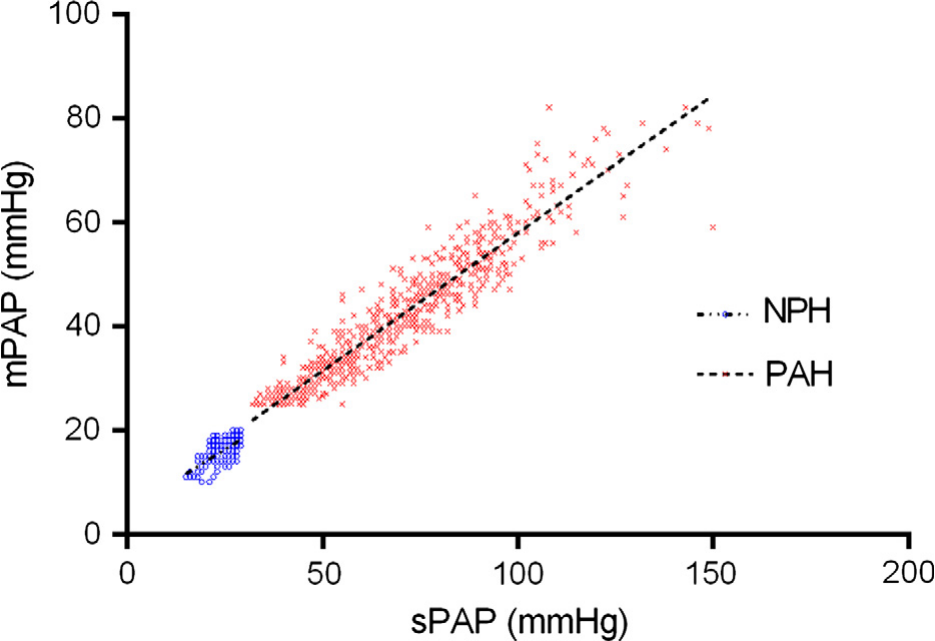
Variable relationship between mPAP and sPAP. NPH: *y*  =  (0.47 × SPAP) + 4.6 (*r*^2^ = 0.50); PAH: *y* = (0.53 × sPAP) + 4.9 (*r*^2^ = 0.89) (*F*-test: *P* < 0.0001).

Analysis of the relationship between mPAP and sPAP reveals two small but significantly different linear relationships (*F*-test: *P* < 0.0001) (Fig.6). The NPH group is best described by mPAP = (0.47 × sPAP) + 4.6 mmHg (*r*^2^ = 0.50). The PAH group is best described by the equation mPAP = (0.53 × sPAP) + 4.9 mmHg (*r*^2^ = 0.89). The combined group is best described by mPAP = (0.55 × sPAP) + 3.5 mmHg (*r*^2^ = 0.94).

This implies (as demonstrated in Table1) that the ratio of mPAP and sPAP is also different between the two groups (*P* < 0.0001). The ratio of mPAP/sPAP correlates with RC time (PVR) (NPH group: correlation value = 0.41, *P* < 0.0001; PAH group: correlation value = 0.53, *P* < 0.0001). The RV oscillatory power fraction is also a function of this ratio (eq. 1).

### Implications for right ventricular function

**Figure 7:**
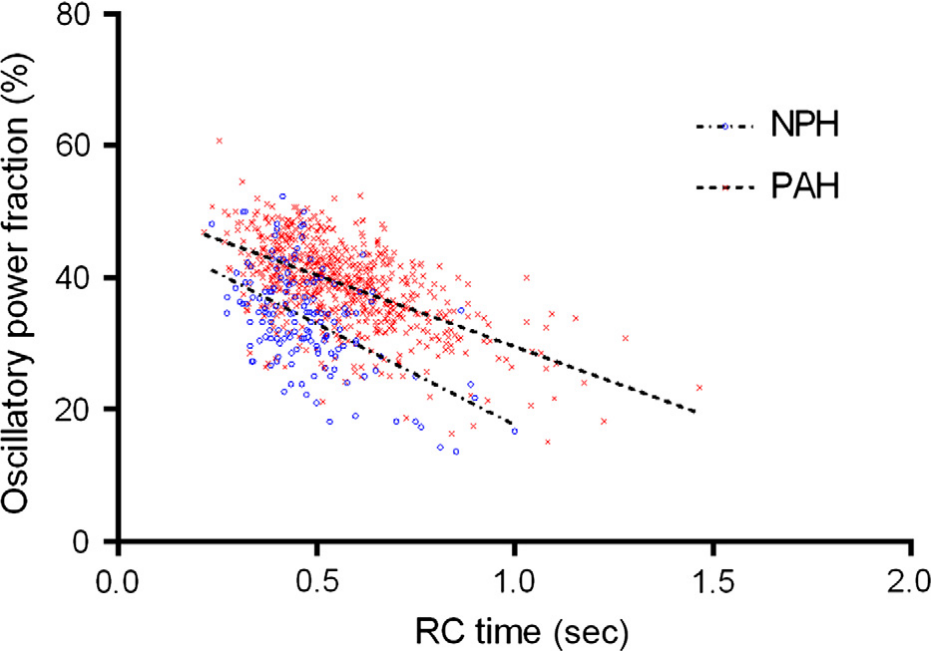
Inverse relationship between RC time and the RV oscillatory power fraction for NPH and PAH groups (NPH: *y* = (−30.8 × RC time) + 48.4 (*r*^2^ = 0.27); PAH: *y* = (−21.7 × RC time) + 51.3 (*r*^2^ = 0.33) (*F*-test: *P* < 0.0001).

RV power parameters for the two groups are summarized in Table2. RV total power, RV mean power, RV oscillatory power, and the RV oscillatory power fraction were all significantly different between the two groups (*P* < 0.0001). There is a negative correlation between RC time and the RV oscillatory power fraction in PAH (Fig.7; correlation value = −0.53, *P* < 0.0001). This implies that a higher proportion of RV power is “wasted” with reduced RC time in PAH.

**Table 2:** Right ventricular power parameters

	NPH group	PAH group	*P*
RV power_total_ (W)	0.25 ± 0.07	0.64 ± 0.24	<0.0001
RV power_mean_ (W)	0.16 ± 0.05	0.39 ± 0.14	<0.0001
RV power_oscillatory_ (W)	0.09 ± 0.03	0.25 ± 0.11	<0.0001
RV oscillatory power fraction (%)	34 ± 7.5	39 ± 6.2	<0.0001

Data presented as mean ± SD. *P*, compared with Wilcoxon rank-sum test. NPH group, normal pulmonary hemodynamics group; PAH group, pulmonary arterial hypertension group; W, watts.

The relationship between RC time and the RV oscillatory power fraction is different between the NPH and PAH groups (Fig.7). Consequently, despite RC time being lower in the NPH group; this group also has a smaller oscillatory power fraction (implying better cardiac efficiency) compared to the PAH group (34 ± 7.5% vs. 39 ± 6.2%, respectively; *P* < 0.0001). There is still a negative correlation between RC time and the RV oscillatory power fraction in the NPH group (correlation value = −0.41, *P* < 0.0001).

The values we report are higher than that reported by Saouti et al. (21% and 23% in their IPAH groups and 25% in their normal pressure group) (Saouti et al. 2010). However, if we apply the correction factor they proposed to take into account that systolic pressure is not constant throughout systole, similar values are obtained (NPH group 21.0 ± 8.9% and PAH group 26.9 ± 7.4%; *P* < 0.0001).

## Discussion

We demonstrate that RC time and the relationship between PVR and Ca is different between health and pulmonary vascular disease. This is supported by a recent analysis by Tedford in which he demonstrates that RC time is lower in subjects with a mPAP <25 mmHg and PVR <3Wood units (Tedford 2014). It has previously been demonstrated that RC time varies with PCWP, in the context of chronic thromboembolic pulmonary hypertension and weakly with age (Tedford et al. 2012; Mackenzie Ross et al. 2013a). However, it is still considered to be a constant outside of these specific situations (Naeije 2013; Tedford 2014). By demonstrating that RC time is significantly different in a group of “all-comers” meeting the hemodynamic definition of PAH compared to hemodynamically “normal” controls we show that RC time is not a constant – rather it varies between health and disease states. Explanations for the lower RC time in low-pressure states include increased measurement error in low-pressure states, a maximal compliance beyond which reducing PVR further does not result in further increases in compliance and possibly the instances in which PCWP is not the downstream determinant of PVR (e.g., when alveolar pressure exceeds PCWP) (Tedford 2014).

RC time is thought to reflect the time constant of the pulmonary circulation. The time constant of biophysical systems, exhibiting exponential behavior in time, is the time taken for a parameter to reduce or increase by 1 – 1/*e* (≈63.2%) of the initial value (Reuben 1971; Laughton and Warne 2003). Therefore, in the pulmonary circulation it is the time for pulmonary artery pressure to decay by 63.2% during the exponential decay phase of diastole (i.e., from the dicrotic notch to PCWP). There remains disagreement whether TPR or PVR-derived RC time values are more physiologically representative of the pulmonary circulation. We demonstrate differences regardless of which method is used. The difference between TPR and PVR-derived RC time is likely a reflection of the influence of PCWP as shown by Tedford et al. 2012. Furthermore, in health PCWP accounts for a larger proportion of mean pulmonary artery pressure.

The RC time, we report, in our normal pulmonary hemodynamics group is 0.47 ± 0.13 sec. Two other studies describe similar groups with normal pulmonary hemodynamics. Tedford et al. describe a group of patients with heart failure (Cohort C Low PCWP group; *n* = 207) (Tedford et al. 2012) and Hilde et al. describe a group with chronic obstructive airways disease with no pulmonary hypertension (*n* = 72) (Hilde et al. 2013). The RC time, we calculate, for these two groups is 0.43 ± 0.30 sec and 0.43 ± 0.27 sec, respectively. This is similar to our own NPH group. Only one other study has directly compared RC time in control subjects (not meeting the hemodynamic definition of PAH) to IPAH and CTEPH patients (Lankhaar et al. 2006). This study used TPR in the calculation of RC time and was not powered to show a difference in RC time (“NONPH” vs. “CTEPH” groups: power = 0.064; “NONPH” vs. “IPAH” groups: power = 0.129).

The subjects with normal pulmonary hemodynamics reported in the three studies described above as well as this current study are not necessarily truly “healthy” given that they have been under investigation for cardiorespiratory symptoms (Lankhaar et al. 2006; Tedford et al. 2012; Hilde et al. 2013). Therefore, we performed a meta-analysis of a systematic review of hemodynamics from healthy volunteers to ascertain RC time in this healthy population (Kovacs et al. 2009). Calculating RC time from this systematic review gives a RC time of 0.39 ± 0.34 sec. This suggests the RC time we report in our “normal” pulmonary hemodynamics group may be an overestimate. We acknowledge limitations in these calculations of RC time using published mean data. This results in potential differences compared to averaging the RC time of individual data points. The effect of this should be minimal in large normally distributed datasets, therefore the assertion that in normal physiology RC time is lower remains valid.

Other groups have previously investigated the effect of therapeutic interventions on RC time and concluded that it remains unchanged (Lankhaar et al. 2008; de Perrot et al. 2011; Skoro-Sajer et al. 2014). Again these studies were not powered to show a difference. de Perrot et al. reported no change in RC time following pulmonary endarterectomy (PEA) in patients with CTEPH (de Perrot et al. 2011). This small study (*n* = 34; data not available for power calculation) used TPR to calculate RC time. As we have demonstrated, TPR-derived RC time is significantly different to PVR-derived values especially in those with normal hemodynamics. Skoro-Sajer et al. reported a nonsignificant fall in RC time following PEA (Skoro-Sajer et al. 2014). We calculate the power of their study, to find a difference in RC time between baseline measurement and 1 year post PEA, is 0.378. A sample size calculation from their data suggests that 386 subjects are required. Furthermore, their data does not distinguish between patients with a predominately proximal distribution of chronic thromboembolic disease and those with more distal disease. We believe that if they were to separate these two groups in their analysis, they would identify patients with proximal disease that are more likely to normalize their hemodynamic post PEA (therefore the sample size required to demonstrate a reduction in RC time decreases). This is an approach we have previously taken and shown a significant decrease in RC time following normalization of hemodynamics post PEA (Mackenzie Ross et al. 2013a). We must now revise our own conclusions regarding the effect of PEA on RC time reported by Mackenzie-Ross et al. Previously, we had suggested that RC time is lower post PEA because of biomechanical changes to the proximal pulmonary arteries as a result of surgery (Mackenzie Ross et al. 2013a). However, with the revised “normal” RC time, it can be concluded that PEA actually normalizes RC time in patients with a good outcome following surgery (defined hemodynamically by a mPAP < 25 mmHg post PEA).

RV oscillatory power does not contribute to the ejection of stroke volume (Saouti et al. 2010). Therefore, the ratio of oscillatory power to total power (the oscillatory power fraction) can be considered as the proportion of “wasted” power. In PAH there is a negative correlation between RC time and the RV oscillatory power fraction (Fig.7). This implies that as RC time reduces, the proportion of “wasted” power is increased – reducing RV efficiency. Therefore, variation in RC time has physiological consequences for RV function. Importantly, the relationship between the RV oscillatory power fraction and RC time was different between the NPH and PAH groups. Consequently, cardiac efficiency was better in our NPH group compared to the PAH group despite their lower RC time. Others have suggested the variation in RC time is small and clinically insignificant due to the small variation in the ratio of total RV power to mean RV power (Naeije and Delcroix 2014). However, our own data suggest that the variation in the RV oscillatory power fraction and consequently the ratio of total to mean RV power is larger.

It should be noted that the gold standard for calculating the RV oscillatory power fraction requires measurement of pulmonary artery flow. Saouti et al. conclude that the RV oscillatory power fraction is a constant based on their study of 46 patients and using fluid-filled catheters as we have done ourselves. They argue that the basis of the constant oscillatory power fraction is due to the universal proportionality between mPAP and sPAP. In our larger dataset we show that this relationship is not the same in the NPH and PAH groups.

This lack of proportionality also has implications for equations predicting mPAP from sPAP. We demonstrate small but significant differences between the best fit equations for the PAH and NPH groups. Previous studies using high-fidelity manometers have suggested there is proportionality between mPAP and sPAP, allowing a universal prediction equation (Chemla et al. 2004, 2009; Syyed et al. 2008). Chemla et al. provide a universal prediction equation of mPAP = (0.61 × sPAP) + 2. This is similar to our own equations. They acknowledge that in narrower hemodynamic ranges and specific patient subgroups alternative equations for predicting mPAP from sPAP maybe more accurate (Chemla et al. 2004).

Concerns have previously been raised regarding the use of fluid-filled catheters rather than high-fidelity manometers. In particular, there are concerns regarding wave reflection and the time response of fluid-filled catheters, especially at higher pressures, in the context of proximal obstructions in CTEPH (Chemla et al. 2013). These issues have been addressed previously (Mackenzie Ross et al. [Bibr b13][Bibr b14]). This study does not use time-dependent hemodynamic parameters, minimizing any errors introduced by the use of fluid-filled catheters. In addition, direct comparison in an animal model of high-fidelity micromanometer tipped catheters and fluid-filled Swan-Ganz catheters (which are used commonly in clinical practice) has shown similar results (Pagnamenta et al. 2013). More recently, Bachman et al. have shown that no clinically significant biases are found when comparing high- fidelity micromanometer tipped catheter and fluid-filled catheter results (Bachman et al. 2013).

The definition of pulmonary compliance must also be taken into consideration. Previous studies have been heterogeneous in their methodology. This has led to significant variations in RC time even within the same disease group and using TPR to derive RC time (Lankhaar et al. 2006; Saouti et al. 2009). The benefits and drawbacks of different methods of calculating Ca have been discussed and their impact on RC time assessed (Lankhaar et al. 2006, 2008). The SV/PP method can be thought as the upper limit of compliance, as it assumes that the proximal pulmonary arteries are exposed to the entire SV, like a closed system (Segers et al. 1999a,b). However, Segers et al. have shown a good correlation between the SV/PP method and the pulse pressure method (considered the gold standard) across several hemodynamic states (Segers et al. 1999a). Additionally, compliance, determined by the SV/PP method, has been correlated with mortality in IPAH and its simplicity may mean it is more accurate than other methods (Mahapatra et al. 2006; Lankhaar et al. 2008). Quantification of pulmonary artery vascular volume may also yield additional information in disease states.

We demonstrate that RC time is not constant between health and diseases with elevated pulmonary artery pressures. Despite this, there remains an inverse relationship between PVR and Ca in all of the studies presented. This relationship is not immutably fixed between health and disease states; although the differences are subtle. We demonstrate that a variable RC time has potential physiological consequences for RV function with a negative correlation with the RV oscillatory power fraction.

## Conflict of Interest

There are no relevant disclosures for any of the authors.
